# Supplementary material for: Time course of visual attention in rats by atomic magnetometer
Source: PLoS One. 2024 Oct 29;19(10):e0312589. doi: 10.1371/journal.pone.0312589 (PMC11521302; doi:10.1371/journal.pone.0312589)
Supplement: S1 File — (DOCX) [file pone.0312589.s001.docx]

Supplementary Information for

**Time course of visual attention in rats by atomic magnetometer**

Authors:

Fan Liu,^1^ Xiang Zhao, ^1^ Yuhai Chen, ^1^ Zhikang Peng, ^1^ Jiahao Wang, ^1^ Jia Yao, ^2^ Ying Zhang, ^3^ Xuejiao Ma,^4^ Qiang Lin^1^ and Yi Ruan^1,*^

Affiliations:

^1^Zhejiang Provincial Key Laboratory and Collaborative Innovation Center for Quantum Precision Measurement, College of Science, Zhejiang University of Technology, Hangzhou 310023, China

^2^Department of Breast Surgery, the First Affiliated Hospital of Zhejiang University School of Medicine. Hangzhou 311121, China

^3^Department of Endocrinology and metabolism, Shaoxing Second Hospital, Shaoxing 312000, China

^4^Beijing Smart-Chip Microelectronics Technology Co. , Ltd. Beijing 102299, China.

* Correspondence to:Yi Ruan, yiruan@zjut.edu.cn

**This PDF file includes:**

Supplementary information Text

Figs. S1

Tables S1

**SERF AM:**

During each measurement, SERF AM parameters were calibrated using a known magnetic field and SERF AM measurements were adjusted within the optimal linear region. The external magnetic field was realized by a pair of Helmholtz coils with a diameter of 5 cm and 150 turns, and its inductance is about 20 mH. The actual measurement shows that the 240 mA current in the coil center region corresponds to a 1.25 mT magnetic field, which was basically consistent with the simulation calculation of COMSOL Multiphysics. FIG.S1 shows the magnetic field distribution in the central plane of parallel and vertical coils respectively.


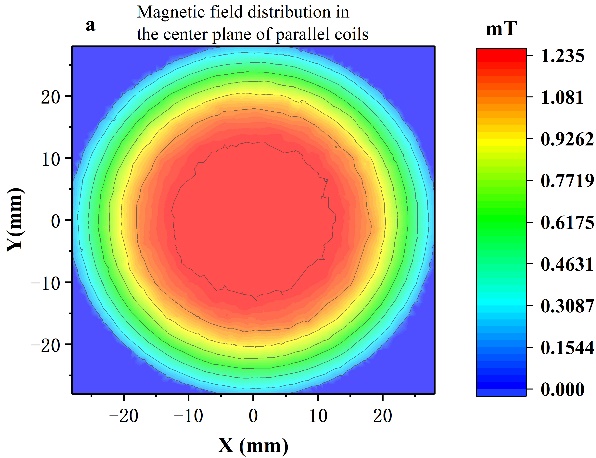

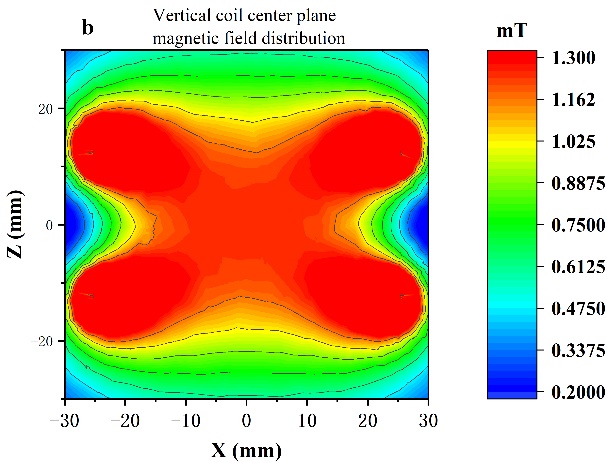


**FIG. S1 Magnetic field distribution of the external magnetic field coils**

|  | Basic experimental information | | | |
| --- | --- | --- | --- | --- |
| Samples | Time interval/s | | | |
|  | 1 | | 2 | |
|  | Amplitude/fT | Incubation Period/ms | Amplitude/fT | Incubation Period/ms |
| 1 | -0.0303 | 91.3` | -0.0361 | 151.7 |
| 2 | -0.0477 | 110 | -0.0161 | 93.4 |
| 3 | -0.0369 | 99 | -0.0275 | 86 |
| 4 | -0.0525 | 97 | -0.0484 | 87.1 |
|  | 3 | | 4 | |
|  | Amplitude/fT | Incubation Period/ms | Amplitude/fT | Incubation Period/ms |
| 1 | -0.0312 | 85.6 | -0.0359 | 21 |
| 2 | -0.02278 | 86.4 | -0.0344 | 51.6 |
| 3 | -0.0472 | 92.1 | -0.0497 | 57.5 |
| 4 | -0.0312 | 85.6 | -0.0342 | 60.4 |
|  | 5 | | 6 | |
|  | Amplitude/fT | Incubation Period/ms | Amplitude/fT | Incubation Period/ms |
| 1 | -0.0199 | 67.2 | -0.0305 | 100.5 |
| 2 | -0.018 | 83.6 | -0.0304 | 100.6 |
| 3 | -0.0307 | 79.1 | -0.0441 | 52.7 |
| 4 | -0.0366 | 123.6 | -0.0701 | 102.5 |
|  | 7 | | 8 | |
|  | Amplitude/fT | Incubation Period/ms | Amplitude/fT | Incubation Period/ms |
| 1 | -0.0947 | 112.2 | -0.1529 | 107.2 |
| 2 | -0.0819 | 106.8 | -0.0931 | 112.1 |
| 3 | -0.1346 | 101.6 | -0.0766 | 111.8 |
| 4 | -0.0769 | 57.6 | -0.107 | 113.3 |

**Table S1. The amplitude and latency of ERMF for each group of samples.**
