# Supplementary material for: Time course of visual attention in rats by atomic magnetometer
Source: PLoS One. 2024 Oct 29;19(10):e0312589. doi: 10.1371/journal.pone.0312589 (PMC11521302; doi:10.1371/journal.pone.0312589)
Supplement: Attachment — Submitted filename: Response to Reviewers.docx [file pone.0312589.s002.docx]

**Reviewer #1:**

**Question 1**: The design method is not introduced in detail, and it feels like a manuscript in the form of a letter rather than a research article. Therefore, the paper needs to give a design method in detail .

**Response 1:** *Thanks for the reviewer’s suggestions. We have added details of our designed method, introduction and conclusion. At page1-2, line 2-54, page 3, line 73-95, page 9, line 213-243.*

**Question 2**: In the result part, there is no data comparison with recent methods, so the innovation of the method cannot be verified. Only the Amplitude and latency of N1 at different ISIs in four rats are given. The paper needs to supplement the data comparison of relevant methods.

**Response 2**: *Thanks for the reviewer’s suggestions. We add the comparison with other methods. At page 1, line 2 , page 3, line 73, page 6, line 167, and page 9, line 213.*

**Reviewer #2:**

**Comments:** This manuscript draft outlines a research study examining the dynamics of visual attention in rats using an atomic magnetometer. The study investigates the relationship between N2-like amplitude and visual attention.

The goal is to measure the N2-like component's amplitude in response to visual stimuli at different inter-stimulus intervals (ISIs) using a passive single-stimulus paradigm. Key findings include:

- The N2-like component's amplitude significantly increases when the ISI exceeds 6 seconds, indicating a sustained attentional state in rats.

- The amplitude decreases with repeated stimuli, showing habituation effects.

- At ISIs of 7 and 8 seconds, the amplitude does not return to baseline levels, suggesting that visual attention refocusing may help maintain the amplitude.

I agree that the study provides valuable insights into the time course of mammalian attention and serves as a reference for future research using the passive single-stimulus paradigm.

**Response:** *We thank Prof. Dr. Murat Özgören for his assessment of our manuscript. The study provides valuable insights into the time course of mammalian attention, specifically in rats, using a passive single-stimulus paradigm. The findings demonstrate that the N2-like component amplitude significantly increases with ISIs exceeding 6 seconds, suggesting a sustained attentional state. The observed decrease in amplitude with repeated stimuli indicates habituation effects. Additionally, the maintenance of amplitude at ISIs of 7 and 8 seconds implies that visual attention refocusing may contribute to sustaining the response amplitude. These results enhance our understanding of visual attention dynamics and offer a reference point for future research in this area.*

*We agree with your suggestion that the terms "intellectualizing" and "remarkable" are inappropriate and have already changed them.*
