# Supplementary material for: Time course of visual attention in rats by atomic magnetometer
Source: PLoS One. 2024 Oct 29;19(10):e0312589. doi: 10.1371/journal.pone.0312589 (PMC11521302; doi:10.1371/journal.pone.0312589)
Supplement: Attachment — Submitted filename: Response to Reviewers-0922.docx [file pone.0312589.s003.docx]

**Reviewer #1:**

**Question 1**:  In the result section, It is suggested that authors add more relevant literature from the last five years for comparative analysis to establish the novelty and significance of the work.

**Response 1:** *Thank you for your comment, we have added a comparison with other articles.*

**Question 2**: The manuscript is well-written, but some small mistakes can benefit form improvement. For example, there are two extra question marks in the sentence line 27 ( impairments??. )

**Response 2**: *Thank you for your comments, we have revised it and checked the full text.*

**Reviewer #2:**

**Response:** *We thank Professor Murat Özgören for his approval of our paper. This study provides valuable insights into the time course of mammalian attention, particularly in rats, using a passive single-stimulus paradigm. The findings showed that the N2-like component amplitude increased significantly when the ISI exceeded 6 seconds, indicating a sustained state of attention. The decrease in amplitude observed upon repeated stimulation suggests the presence of a habituation effect. Furthermore, the maintenance of amplitude at ISIs of 7 and 8 s implies that refocusing of visual attention may contribute to the maintenance of response amplitude. These results enhance our understanding of the dynamics of visual attention and provide a reference point for future research in this area.*
